# Two Schemes in Hereford and Suffolk

**Published:** 1920-06-19

**Authors:** 


### AGRICULTURAL CONTRIBUTIONS.

### Two Schemes in Hereford and Suffolk.

There seems to be a genuine attempt to organise the most important of home industries, namely, agriculture, in support of the voluntary hospitals. At the same moment we learn of activity in Herefordshire and activity in Suffolk. The farmers of Herefordshire have started a County Farmers' Hospital League for the benefit of Hereford Hospital, at a recent meeting at the Palace, Hereford, when the Bishop of Hereford made an excellent speech and told the farmers more than one interesting thing about the agriculturists' character. Farmers, the Bishop said, were the backbone of the county, and therefore so important a county institution as the hospital ought to have the farmers' loyal and steady support. In 1919, so his information went, only fourteen farmers subscribed. Yet his experience was that farmers were not ungenerous, but their association caused them to be leisurely in making up their minds. Hereford hospital, however, was founded in 1776, so that the farmers had had sufficient time to adopt the idea of supporting it. Farmers were slow to part with ready money, but generous in other ways. He had heard of prosperous farmers in the north who would not give a guinea to anything, but would give an ox or a sheep, which was worth much more. He urged them, therefore, to join the league and subscribe a guinea, and then help the committee of the hospital with gifts of stock, and organise a Farmers' Sale for its benefit. The Cottagers' Organisation, under the Cresswell Penny Fund, last year raised £661, the farmers should raise a sum in proportion to their importance in the county.

A County Farmers' Hospital League was formed with an active farmer as president. The different districts are to be represented on the executive committee.

The Suffolk Agricultural Association met at Bury St. Edmunds, on almost the same day as the Hereford meeting; the Right. Hon. E. G. Pretyman, M.P., presided. His idea is parallel to that put forward at Hereford, but not identical with it. Why, he asked, should not agricultural labour follow the lead of industrial labour and subscribe by voluntary organisation one penny a week per man? On certain farms in Suffolk, we understand, this plan was adopted till compulsory insurance at a time when agricultural wages were low led to a stoppage of these voluntary contributions. To exclude institutional treatment made the Act from the first an expensive farce to insured persons. Now that wages have risen can these voluntary gifts to Suffolk hospitals be revived, and will the farmers do their share by contributing a penny for each weekly penny given by their labourers?

This was Mr. Pretyman's suggestion. He had sounded the farmers' organisations, the labour organisations, and the owners, who were in favour of the scheme. He suggested a conference between the heads of the farmers' and labourers' organisations with the committees of the hospitals in their areas. He hoped much from the scheme, which should be applied to all forms of industry.

Since writing the above, Mr. B. B. Taylor has pointed out that country workers include teachers and schoolchildren, and that at the school at Capel St. Mary, from which he wrote, a scheme of hospital support has been started. The Scripture lesson includes references to hospital work, and the children have agreed to contribute ¼d. per week or 1d. per month. The total is sent to the hospital, earmarked for the children's ward. The collecting-box is opened by one of the elder scholars once a month; the money is put in at odd times, and no one knows who contributes. This school expects to raise £6 a year, and, as there are said to be 200 schools in East Suffolk, an income of £1,000 could be raised were the plan generally adopted.

